# Top-Down LESA Mass Spectrometry Protein Analysis of Gram-Positive and Gram-Negative Bacteria

**DOI:** 10.1007/s13361-017-1718-8

**Published:** 2017-07-05

**Authors:** Klaudia I. Kocurek, Leanne Stones, Josephine Bunch, Robin C. May, Helen J. Cooper

**Affiliations:** 1 School of Biosciences, University of Birmingham, Edgbaston, Birmingham, B15 2TT UK; 2 Institute of Microbiology and Infection, University of Birmingham, Edgbaston, Birmingham, B15 2TT UK; 3 National Physical Laboratory, Hampton Road, Teddington, TW11 0LW UK; 4 School of Pharmacy, University of Nottingham, University Park, Nottingham, NG7 2RD UK

**Keywords:** Top-down protein analysis, LESA, Ambient mass spectrometry, Gram-positive, Gram-negative

## Abstract

We have previously shown that liquid extraction surface analysis (LESA) mass spectrometry (MS) is a technique suitable for the top-down analysis of proteins directly from intact colonies of the Gram-negative bacterium *Escherichia coli* K-12. Here we extend the application of LESA MS to Gram-negative *Pseudomonas aeruginosa* PS1054 and Gram-positive *Staphylococcus aureus* MSSA476, as well as two strains of *E. coli* (K-12 and BL21 mCherry) and an unknown species of *Staphylococcus*. Moreover, we demonstrate the discrimination between three species of Gram-positive *Streptococcus* (*Streptococcus pneumoniae* D39, and the viridans group *Streptococcus oralis* ATCC 35037 and *Streptococcus gordonii* ATCC35105), a recognized challenge for matrix-assisted laser desorption ionization time-of-flight MS. A range of the proteins detected were selected for top-down LESA MS/MS. Thirty-nine proteins were identified by top-down LESA MS/MS, including 16 proteins that have not previously been observed by any other technique. The potential of LESA MS for classification and characterization of novel species is illustrated by the de novo sequencing of a new protein from the unknown species of *Staphylococcus*.

**Electronic supplementary material:**

The online version of this article (doi:10.1007/s13361-017-1718-8) contains supplementary material, which is available to authorized users.

**Graphical Abstract:**
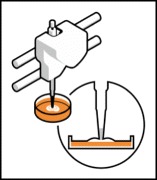
ᅟ

## Introduction

The application of mass spectrometry (MS) for the identification and characterization of bacterial species has a history of more than 40 years [1]. It has emerged as a mature and powerful technique for the study of microorganisms, shedding light on the chemical composition of their cells and colonies, from metabolites through lipids to entire proteomes.

Detection of characteristic compounds in bacterial samples allows their identification down to a genus or, in rarer instances, species and strain level [2]. Matrix-assisted laser desorption ionization (MALDI) time-of-flight (TOF) MS, the most commonly used technique [3], has been in development for that purpose since the late 1990s [4, 5] (recently reviewed in [6]). It relies primarily on spectral fingerprinting—matching mass spectra acquired from unknown samples against databases filled with mass spectra of previously identified microorganisms. Thanks to its long development time and resulting robustness, it is currently being rolled out worldwide as a clinical and diagnostic tool with the potential to eventually replace classic microbiological identification methods [7, 8], although discrimination between certain strains and species (e.g., *Escherichia coli* and *Shigella* or the viridans group streptococci) remains a challenge for the current protocols [6].

The MALDI-TOF approach does, however, have drawbacks that limit its use for the characterization of proteins from microbial colonies and biofilms. The most significant obstacle lies in sample preparation for analysis in a vacuum; whereas the routine preparation of bacterial smears for the purposes of clinical identification is trivial, the study of intact biofilms directly on growth media has proven challenging. Even though multiple groups have developed protocols for MALDI imaging analysis of bacterial colonies on agar [9–12], there is no consistency in the sample preparation methods across the literature, even where identical equipment has been used. The problems reported include insufficient matrix saturation, uneven coverage, and flaking of the sample under a vacuum, the latter issue being by far the most serious as it may occasionally lead to instrument damage [12].

The challenge of sample preparation can be eliminated by use of ambient ionization techniques, although frequently at the cost of the capability for protein detection. Desorption electrospray ionization (DESI) has been applied to the analysis of bacterial suspensions dried onto solid substrates [13, 14], and colonies grown on filter membranes [15]; however, direct measurements on agar could not be easily obtained without drying of the sample because of the soft and conductive nature of the nutrient medium [16]. Rapid evaporative ionization MS has been extensively tested for the characterization of lipid profiles directly from cultured bacterial colonies, and as a high-throughput identification platform [17–19]. Laser ablation electrospray ionization has been used to directly probe biofilms on filter paper, providing information largely limited to lipids and metabolites [16, 20]. Similarly, paper spray MS of bacterial colony smears, generating mass spectra primarily consisting of phospholipid peaks, was shown to provide enough information for bacterial identification at the species level [21]. NanoDESI and the liquid microjunction surface sampling probe, both making use of liquid microjunction extraction, have been shown to extract small (up to 4.5 kDa), secreted peptides as well [22, 23].

One related liquid microjunction method coupled with high-resolution MS, liquid extraction surface analysis (LESA), has consistently proven to be suitable for protein extraction. To date, it has been used to characterize a wide array of samples, ranging from dried blood spots [24–27] through tissue sections [28, 29] to living bacterial colonies [29, 30]. A straightforward, solvent-based technique, suitable for manual application although usually performed by use of the dedicated TriVersa NanoMate automatic pipette system [31], LESA relies on the deposition of a droplet of solvent on the surface to be sampled. The droplet is held in contact with the surface, forming a liquid microjunction through which analytes diffuse into the solvent. The droplet is then withdrawn into the electroconductive pipette tip and introduced into the mass spectrometer by nanoelectrospray ionization. The distinct advantage of LESA is the ability to simultaneously extract multiple types of analytes from a single location with little to no sample preparation. This versatility means that a single instrument could potentially be capable of analyzing a vast range of clinically relevant sample types for different biomarkers. The extraction of multiple classes of molecules may in some cases produce overly complex mass spectra or lead to extensive ion suppression [32]; to circumvent this issue, separation methods such as nano liquid chromatography (LC)–MS [28] or high-field asymmetric waveform ion mobility separation (FAIMS) [26, 29] may be used before MS analysis.

We have previously reported top-down LESA MS identification of proteins extracted directly from colonies of *E. coli* K-12 grown on agar plates [29, 30]. Seven proteins were identified: three DNA-binding proteins and four stress response proteins, one of which required FAIMS separation of the sample to be detected. Crucially, the protein signal could not be observed unless a modified protocol, bringing the tip of the sampling pipette in contact with the colony surface (“contact” LESA), was used. It has been suggested that the inability to detect proteins when standard LESA is used arises from the presence of the extracellular matrix around and within the colony that requires physical disruption to allow the extraction of proteins from the bacterial cells.

Here we present the application of this approach to two clinical isolate species, the Gram-negative *Pseudomonas aeruginosa* PS1054 and the Gram-positive *Staphylococcus aureus* MSSA476, as well as three representatives of a group of closely related species of Gram-positive streptococci, known to cause nonidentification and misidentification issues in clinically deployed MALDI-TOF MS systems [33, 34], and an unknown laboratory strain of *Staphylococcus*. Two strains of *E. coli*, K-12 and BL21 labeled with mCherry, were also analyzed. The results show the broad applicability of LESA MS to the analysis of proteins in bacterial colonies. Importantly, differentiation of the streptococci was straightforward by LESA MS. Top-down LESA MS is demonstrated by the identification of nearly 40 proteins, more than 40% of which have not been observed previously by any other biochemical or MS technique. De novo identification of a further protein from an unknown *Staphylococcus* species is also demonstrated, illustrating the potential of LESA MS for classification and characterization of novel species.

## Experimental

### Materials

*E. coli* K-12, *E. coli* BL21 mCherry, *P. aeruginosa* PS1054, *S. aureus* MSSA476, and an unidentified *Staphylococcus* species were cultured on solid Lysogeny broth agar (LBA) medium in 6-cm-diameter petri dishes. (The size of the plates is limited by the size of the sampling tray that forms part of the Advion TriVersa NanoMate system.) *Streptococcus pneumoniae* D39, *Streptococcus oralis* ATCC 35037, and *Streptococcus gordonii* ATCC 35105 were cultured on blood agar either in open air or under semianaerobic conditions by use of a candle jar. All strains were incubated at 37 °C for 24–48 h and stored at room temperature or 4 °C if necessary; deliberate deviations from the established protocols are noted in the text.

The solvent system used for liquid extraction consisted of acetonitrile (J.T.Baker, Deventer, Netherlands), water (J.T.Baker, Netherlands), and formic acid (Sigma-Aldrich, Gillingham, UK); the relative proportions were either 39.5:59.5:1 or 40:60:1 for Gram-negative species, and either 40:60:1 (for high nanoelectrospray stability, allowing acquisition of tandem mass spectra) or 50:45:5 (for rapid, reliable generation of full mass spectra) for Gram-positive species as noted.

### Liquid Extraction Surface Analysis

Petri dishes containing colonies of interest were placed in the sample tray of a TriVersa NanoMate robot (Advion, Ithaca, NY, USA), adjacent to a quarter of a 96-well microtiter plate holding the solvent system. The position of the colonies was indicated with use of the advanced user interface (AUI) of the program ChipSoft controlling the movements of the robotic pipette. Solvent (3 μl) was aspirated from a well of the microtiter plate. The pipette tip was moved above the colony to the position specified by the advanced user interface and brought into contact with the colony surface by the lowering of it to a height of approximately -10 mm; the exact distance would be adjusted for each individual agar plate and reassessed periodically to account for the gradual changes in the height of the solid agar medium. On contact, 2 μl of the solvent was dispensed onto the surface of the colony encompassed by the pipette tip. Contact was maintained for a minimum of 3 s, following which the solution was reaspirated into the pipette tip.

LESA MS was undertaken in an Advisory Committee on Dangerous Pathogens Containment Level 2 laboratory. No viable bacterial cells are present in the LESA droplet following sampling. To verify that, solvent droplets following LESA extraction from live bacterial colonies were spotted onto agar plates and incubated overnight. No growth was observed (see Supplementary File 1).

### Mass Spectrometry

All MS experiments were performed with an Orbitrap Elite instrument (Thermo Fisher Scientific, Bremen, Germany) at a resolution of 120,000 at *m*/*z* 400. Samples were introduced into the instrument by use of the TriVersa NanoMate integrated, chip-based nanoelectrospray system at a pressure of 0.3 psi and a voltage of 1.75 kV. Full-scan mass spectra in the *m*/*z* range from 600 to 2000 were acquired for a minimum of 5 min. Each scan comprised ten coadded microscans. Precursor ions were selected for fragmentation with an isolation window of 2, 3, or 5 *m*/*z* as appropriate. Collision-induced dissociation was performed in the ion trap with use of helium gas at a normalized collision energy of 35%, and the fragments were detected in the Orbitrap. MS/MS spectra were recorded for 5 min. Each MS/MS scan comprised 30 coadded microscans. Automatic gain control targets were 1 × 10^6^ charges for full-scan mass spectra and 5 × 10^5^ charges for MS/MS spectra.

### Protein Identification

Top-down protein identification was performed with ProSight 3.0 software (Thermo Fisher Scientific, Bremen, Germany). MS/MS spectra were deconvoluted by THRASH at a signal-to-noise ratio of 3. These were then matched against custom databases constructed for each species from full proteome data available via UniProt.

Each database was constructed as a standard top-down database, taking into account the cleavage of initial methionines and N-terminal acetylation but ignoring formylation. SNPs and all available posttranslational modifications were considered, with up to 13 features per sequence and a maximum mass of 70 kDa. For both strains of *E. coli*, the complete, annotated reference proteome of the K-12 strain was used (UniProt ID UP000000625, 4306 protein entries). For *S. aureus* MSSA476, two databases were generated: one based on the reference proteome of a representative strain (NCTC 8325, UniProt ID UP000008816, 2889 protein entries), making use of the more complete, reviewed annotation, and one specific to the MSSA476 strain (UniProt ID UP000002201, 2598 protein entries). For *P. aeruginosa* PS1054 the reference proteome (UniProt ID UP000002438, 5563 protein entries) was used exclusively. *S. pneumoniae* D39 was searched against the reference proteome of a closely related, nonpathogenic strain (UniProt ID UP000000586, 2030 protein entries). The *S. oralis* database was based on the only available reference proteome for this species (UniProt ID UP000005621, 2022 protein entries). Two *S. gordonii* databases were used, one based on the annotated chromosome (UniProt ID UP000001131, 2050 protein entries) and one based on the whole genome sequence (UniProt ID UP000069207, 2095 protein entries). A multispecies database was also constructed based on the pan-proteomes of *S. pneumoniae* ATCC BAA-255/R6 (UniProt ID UP000000586) and *S. gordonii* Challis (UniProt ID UP000001131; a total of 61,367 protein entries). Tandem mass spectra of the unknown species of *Staphylococcus* were searched against the reference *Staphylococcus epidermidis* proteome (UniProt ID UP000000531, 2492 protein entries) and a concatenated multispecies database comprising all available nonredundant proteomes in the *Staphylococcus* genus (145,289 protein entries), referred to as the “all *Staphylococcus*” database in the text.

For each database, a broad absolute mass search was specified as a starting point—proteins were considered within 2 kDa of the measured intact mass, with both Δ*m* and disulfide modes active and all available posttranslational modifications taken into account; fragment tolerance was set to ±15 ppm. Putative hits, as well as any posttranslational modifications, were verified by narrowing down the search criteria and optimizing any remaining matches in ProSight’s Sequence Gazer, followed by manual peak assignment with an acceptance threshold of ±5 ppm.

The protein sequence generated de novo from the unknown *Staphylococcus* species was searched by standard protein BLAST against all nonredundant protein sequences belonging to the *Staphylococcus* genus (taxonomic ID 1279), with use of the protein–protein BLAST algorithm with the default BLOSUM62 scoring matrix.

## Results and Discussion

### LESA Sampling of Bacteria

The successful generation of protein mass spectra following LESA sampling of bacteria appears to be dependent on the physical properties of the colonies, in particular the tendency of colony material to adhere to the pipette tip, as well as colony size and surface variability. *E. coli* K-12, the model bacterium used to first develop the protocol and chosen to measure sampling reproducibility, was also by far the most challenging from which to successfully generate protein mass spectra. Initial experiments comprising a total of 125 sampling attempts across colonies grown and stored under a range of conditions yielded 62 mass spectra, of which 31 contained a clear protein signal, an overall success rate of 25%. In the later refrigeration experiments (see below), the success rate was 49% (30 protein mass spectra from 61 sampling attempts). The failure to acquire mass spectra following sampling was universally due to the nanoelectrospray nozzles becoming blocked with aspirated colony matter. A careful choice of sampling height (touching rather than piercing the colony) alleviated the issue although it is currently limited by the minimum vertical step size of 0.2 mm on the TriVersa NanoMate robot.

*Pseudomonas* colonies were less sensitive to the choice of sampling height, reliably generating protein-rich mass spectra. For example, in the refrigeration experiments described later, 45 sampling attempts resulted in 37 protein mass spectra (82% success rate). The Gram-positive staphylococci were similarly amenable to sampling, although rather than cytosolic proteins, they yielded mainly secreted proteins. Initial experiments with the staphylococci using the same solvent system as for the Gram-negative species (i.e., 40:60:1 acetonitrile–water–formic acid) had a success rate of approximately 50%. Optimization of the solvent system (to 50:45:5 acetonitrile–water–formic acid) increased the sampling success rates, possibly due to increased extraction as a result of cell lysis and/or increased ionization efficiency. With this solvent system, the sampling success rate in the refrigeration experiments was 97% (28 protein mass spectra obtained from 29 sampling attempts). The same solvent was applied to the sampling of Gram-positive streptococci.

### General Overview of the Mass Spectra

**Figure 1:**
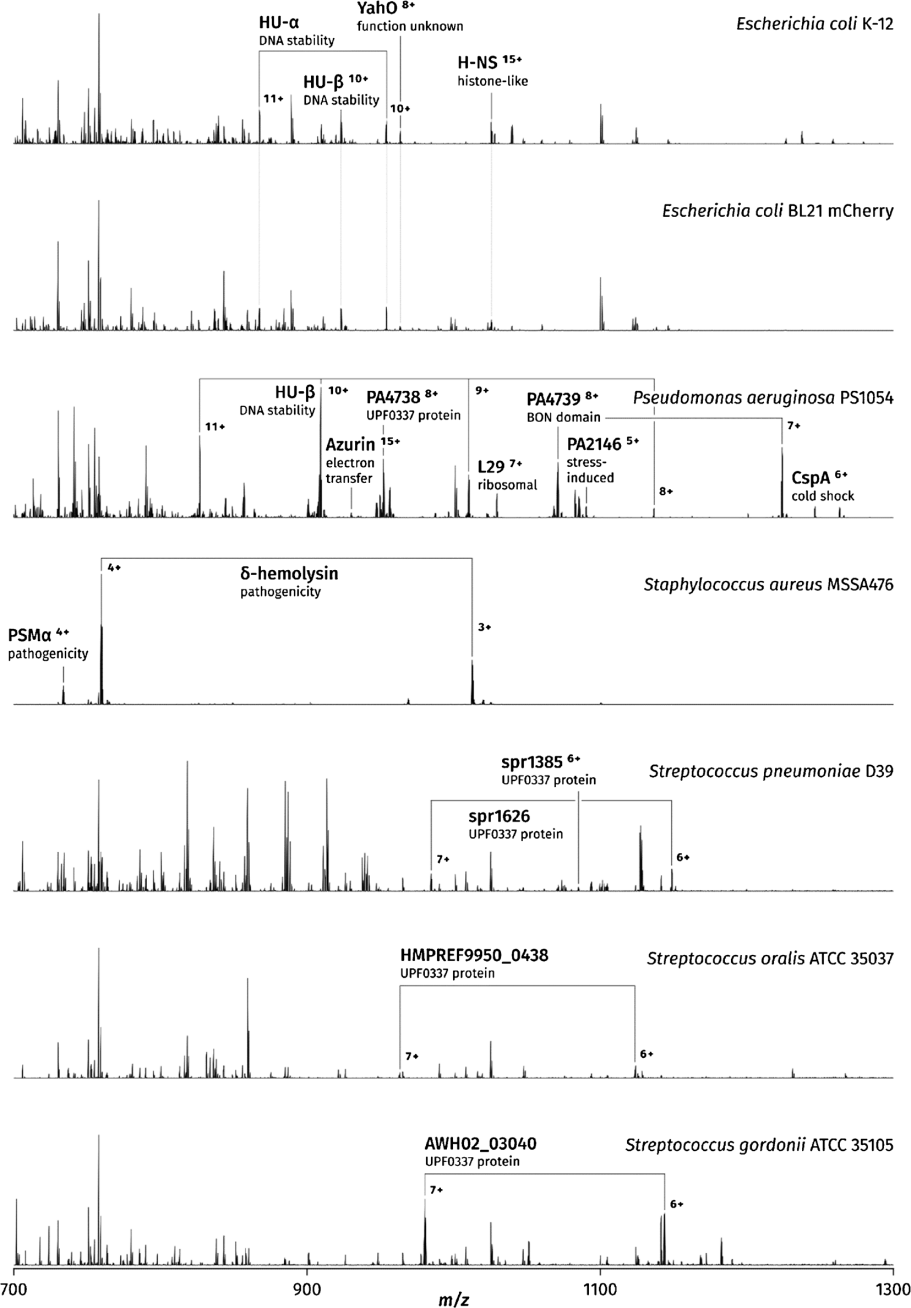
Liquid extraction surface analysis (LESA) mass spectra of seven bacterial strains: *Escherichia coli* K-12, *Escherichia coli* BL21 mCherry, *Pseudomonas aeruginosa* PS1054, *Staphylococcus aureus* MSSA476, *Streptococcus pneumoniae* D39, *Streptococcus oralis* ATCC 35037, and *Streptococcus gordonii* ATCC 35105. All mass spectra were acquired immediately following incubation of the colonies at 37 °C, with use of the 40:60:1 acetonitrile–water–formic acid solvent system for Gram-negative bacteria and the 50:45:5 variant for Gram-positive bacteria. The streptococci were incubated under semianaerobic conditions for optimum growth; the remaining strains were grown in open air

Figure 1 shows representative mass spectra of seven bacterial colonies corresponding to seven strains: Gram-negative *E. coli* K-12, *E. coli* BL21 mCherry, and *P. aeruginosa* PS1054, and Gram-positive *S. aureus* MSSA476, *S. pneumoniae* D39, *S. oralis* ATCC 35037, and *S. gordonii* ATCC 35105. All colonies were sampled immediately following incubation at 37 °C, with use of either the 40:60:1 acetonitrile-based solvent system for Gram-negative bacteria or the 50:45:5 variant for Gram-positive bacteria.

*P. aeruginosa* gave rise to highly reproducible mass spectra, particularly easy to acquire and rich in protein peaks. Prominent, consistent protein peaks corresponding to five proteins appeared in every acquired mass spectrum. The total number of protein peaks observed in a representative mass spectrum generated following use of optimum incubation conditions was 66, corresponding to 20 unique masses, excluding suspected adducts and modifications. Fifteen of these were selected for MS/MS identification (see later). The total number of protein peaks in a representative spectrum of *E. coli* was 44, corresponding to 14 unique masses; alteration of the growth conditions, such as subjecting the colonies to cold or room temperature storage, increased the number of unique masses to at least 19 by inducing the expression of multiple stress response proteins (see later). The set of predictable protein peaks in *E. coli* corresponded to the DNA-binding proteins HU-α and HU-β in up to four charge states. Whereas the number of observed proteins is markedly lower than the 150+ proteins reported by top-down LC–MS of *E. coli* K-12 extracts [35], the acquisition of data by LESA MS is much faster and more straightforward as it involves no sample preparation nor lengthy separation before analysis. Addition of a separation step, such as FAIMS [29] or LC [27], into the LESA MS workflow is possible, and would likely expand the range of detected proteins.

Crucially, direct comparison of LESA mass spectra acquired from the two Gram-negative species revealed considerable promise for the technique to be used as an in situ microbial identification tool. Although the signal at the lower end of the *m*/*z* range was similar for both species, because of the presence of peaks derived from the Lysogeny broth agar (LBA) growth medium as well as common lipids and metabolites, unambiguous differentiation between the mass spectra was still possible as the range of protein peaks found in each species was unique.

The mass spectra of *E. coli* K-12 and *E. coli* BL21 mCherry were highly similar. Peaks corresponding to the characteristic DNA-binding proteins HU-α, HU-β, and H-NS as well as the putative periplasmic protein YahO were seen in mass spectra of both strains; numerous low-mass species were also common to both strains and occurred at similar abundances. Whereas the BL21 strain should be differentiated by the presence of the 28.8-kDa mCherry fluorescent protein, no peaks that could be confidently assigned to this protein were observed.

The mass spectra of *S. aureus* were dominated by peaks corresponding to secreted peptides, the most abundant of which was the well-characterized toxin δ-hemolysin. A representative mass spectrum acquired with 5% formic acid, as seen in Figure 1, contained 26 peaks corresponding to 11 unique protein and peptide masses.

### Differentiation of Streptococci

As an additional challenge, colonies of three species of *Streptococcus*—*S. pneumoniae* D39, *S. oralis* ATCC 35037, and *S. gordonii* ATCC 35105—were subjected to LESA MS characterization. Streptococci are encapsulated Gram-positive bacteria, seen in nature both as pathogens and as harmless commensals. *S. pneumoniae* and the viridans group streptococci (including *S. oralis* and *S. gordonii*, which constitute a major part of dental plaque) are of particular clinical interest, as the current method for their differentiation, which is required for the identification of a correct treatment, relies primarily on an optochin test and similar microbiological techniques. Distinguishing these two groups of streptococci by MALDI-TOF MS presents a challenge because of the high similarity of their fingerprint mass spectra; the performance of any individual identification platform in this task is largely dependent on the spectral databases used [3, 33, 34]. An unambiguous identification approach based on defined, individual peaks may therefore prove superior.

The thick capsule surrounding streptococcal cells was expected to hinder lysis and therefore reduce the efficiency of protein extraction. Predictably, the protein signal generated from the colonies was much weaker than in the case of all other investigated strains; 1% and 5% formic acid solvents were both used, with no marked improvement observed at the higher formic acid concentration. Nevertheless, eight peaks corresponding to five protein masses were detected in *S. pneumoniae*, and nine peaks corresponding to six protein masses were detected in *S. oralis*; *S. gordonii* gave rise to richer mass spectra, with 26 peaks and 15 protein masses detected. Only one protein (observed mass 7984 Da) was common to all three species; all other protein masses observed were unique to their species. That is, differentiation of these species on the basis of their LESA mass spectra was straightforward. Four proteins were selected for MS/MS identification; none of these have thus far been directly observed as intact proteins (for details see the following section). Fragmentation data were searched against a combined protein database comprising multiple strains of *S. pneumoniae*, *S. oralis*, *Streptococcus mitis*, *Streptococcus pseudopneumoniae*, *S. gordonii*, and other closely related species. All four proteins were confidently assigned (expectation value less than 1 × 10^-70^) to the correct species.

### Top-Down Protein Identification

Table 1 lists the proteins identified in all bacterial strains investigated. Fragment assignments are given in Supplementary File 2. Sample growth and storage conditions were deliberately varied, allowing the observation of a wider range of proteins, particularly ones involved in stress response; the conditions under which the tandem mass spectrum of any given protein was acquired are listed in Table 1 (although these were not necessarily the only conditions under which the protein was observed). Notably, numerous proteins reported here have not been directly observed in their intact form by any other technique. Novel protein identifications are shown in bold type in Table 1.

**Table 1:** Proteins Identified by Liquid Extraction Surface Analysis Mass Spectrometry in Sampled Colonies

*m*/*z*	Charge	Molecular weight observed	Mass difference (ppm)	ID	UniProt accession no.	Cov (%)	Conditions	Modifications
*Escherichia coli* BL21								
959.1288	+6	5748.73	-0.8	YmdF	P56614	66	Incubation: 24 h, 37 °C Storage: 4 days, room temperature	-Met
978.9706	+6	5867.78	-2.0	YciG	P21361	36		-Met
1101.2759	+7	7701.88	-1.5	YahO	P75694	91		-signal peptide
1189.5904	+7	8320.08	-1.3	UPF0337 protein YjbJ^a^	P68206	80		
1254.2614	+7	8772.78	-2.2	YdfK	P76154	31		
1494.9477	+7	10,457.58	+1.02 Da	**YbgS**	P0AAV6	45		-signal peptide; putative disulfide 71–76 and deamidation
*Escherichia coli* K-12								
923.0049	+10	9219.98	-2.0	HU-β^a^	P0ACF4	43	Incubation: 24 h, 37 °C Storage: 2 days, room temperature	
953.9263	+10	9529.93	0.2	HU-α^a^	P0ACF0	40	Incubation: 24 h, 37 °C Storage: 15 days, 4 °C	
963.5065	+16	15,399.99	+1.02 Da	H-NS^a^	P0ACF8	30	Incubation: 24h, 37 °C Storage: 2 days, room temperature	-Met
1019.5584	+5	5092.76	-1.9	SRA	P68191	11		
1039.4342	+7	7268.99	0.8	L29	P0A7M6	41	Incubation: 24 h, 37 °C Storage: 15 days, 4 °C	
1088.8935	+6	6527.32	-1.1	BhsA^a^	P0AB40	35		-signal peptide
1094.2255	+8	8745.75	-0.6	YnaE	P76073	62		
1212.1269	+6	7266.72	-0.4	CspC	P0A9Y6	37	Incubation: 24 h, 37 °C Storage: 2 days, room temperature	-Met
1356.6814	+3	4067.02	0.3	CydX	P56100	46	Incubation: 24 h, 37 °C Storage: 18 days, 4 °C	fMet
*Pseudomonas aeruginosa* PS1054								
672.2674	+9	6041.34	-1.8	L33	Q9HTN9	31	Incubation and storage: 4 days, room temperature	
723.8214	+10	7228.14	-2.0	**L35**	Q9I0A1	41		-Met
739.5860	+6	4431.47	-1.2	**L36**	Q9HWF6	45		
759.9734	+11	8348.63	-1.4	S21	Q9I5V8	29		-Met
826.5565	+11	9081.04	-0.3	HU-β	P05384	51	Incubation: 48 h, 37 °C Sampled fresh	
951.8583	+8	7606.81	0.0	UPF0337 protein PA4738	Q9HV61	58		
956.3376	+6	5731.98	0.1	**PA0039**	Q9I793	24	Incubation: 24 h, 37 °C Sampled fresh	-signal peptide, 4–42 disulfide
958.5127	+16	15,320.09	3.5	**PA5178**	Q9HU11	27	Incubation: 48 h, 37 °C Sampled fresh	-Met
983.5923	+10	9825.85	-1.6	**Peptidylprolyl isomerase**	Q9HWK5	45	Incubation: 24 h, 37 °C Storage: 4 days, room temperature	-Met
990.3747	+8	7914.94	-1.4	L31	Q9HUD0	31		
996.4895	+14	13,936.75	-0.8	Azurin	P00282	14		-signal peptide, disulfide
1029.1389	+7	7196.92	-0.6	L29	Q9HWE3	29	Incubation: 48 h, 37 °C Sampled fresh	
1090.1381	+5	5445.65	0.4	**PA2146**	Q9I1W9	67		-Met
1223.5075	+7	8557.50	-0.7	**PA4739**	Q9HV60	61		-signal peptide (1–32)
1245.9733	+6	7469.80	-0.2	**CspA**	P95459	31		-Met
*Staphylococcus aureus* MSSA476								
733.7894	+3	2198.35	0.6	Phenol-soluble modulin α_4_	P0C826	100	Incubation: 24 h, 37 °C Sampled fresh	fMet
759.6682	+4	3034.64	0.8	δ-Hemolysin	Q9I5V8	100	Incubation: 48 h, 37 °C Sampled fresh	fMet
921.1190	+6	5520.67	2.0	**Uncharacterized protein SAOUHSC_01729**	Q2FXV2	96	Incubation: 24 h, 37 °C Sampled fresh	
1136.8509	+4	4521.39	2.0	**Uncharacterized protein SAOUHSC_01135**	Q2FZA4	82		fMet, sodium adduct
1148.2490	+6	6883.45	1.7	**UPF0337 protein SAOUHSC_00845**	Q2FZY9	89		
*Streptococcus pneumoniae* D39								
930.0688	+7	6503.43	0.3	**UPF0337 protein spr1385**	Q8CYJ5	85	Incubation: 24 h, 37 °C Sampled fresh	-Met
984.6548	+7	6884.53	0.4	**UPF0337 protein spr1626**	Q8CYD7	76		
*Streptococcus oralis*								
963.0736	+7	6734.46	-2.0	**UPF0337 protein HMPREF9950_0438**	F9Q496	82	Incubation: 24 h, 37 °C Sampled fresh	-Met
*Streptococcus gordonii*								
980.2427	+7	6854.65	-0.6	**UPF0337 protein AWH02_03040**	A0A0F5MKW3	71	Incubation: 48 h, 37 °C, semianaerobic Sampled fresh	-Met

Protein names in bold signify proteins that have been directly observed for the first time.

*fMet* formylmethionine, *Met* methionine

^a^Proteins identified in the previous study on *Escherichia coli* K-12 using liquid extraction surface analysis mass spectrometry

*P. aeruginosa* was arguably the most amenable to LESA MS analysis among the species chosen, owing to the fast growth of colonies, the ease of sampling, and the wide range of detected proteins. Several of those, such as the DNA-binding protein HU-β or the ribosomal constituent L29, were homologs of proteins detected in *E. coli*. PA2146, a KGG stress response motif protein predicted from the full genome sequence [36], is noteworthy as a relative of *E. coli* YciG and YmdF, both newly identified here. Most notably, however, the UPF0337 family of stress response proteins, represented in *P. aeruginosa* by PA4738, was observed both in *E. coli* (YjbJ) and in *S. aureus* (SAOUHSC_00845) as well as in all three streptococci.

In addition to PA2146, *P. aeruginosa* yielded multiple proteins whose existence is predicted on the basis of genome data, in several instances bolstered by the presence of homologous sequences in other species, but which have not previously been detected as proteins. A prime example of such a protein was peptidylprolyl isomerase PpiC2, a small predicted enzyme related to *E. coli* parvulin [36]; PpiC2 was identified here in its intact form, missing the N-terminal methionine. PA4739, another predicted protein detected by LESA MS, contains a putative BON domain, which suggests it is localized to either the periplasm or the outer membrane, and is likely involved in stress response [37]. Automatic gene annotation by UniProt (accession number Q9HV60) suggests it contains a 25 amino acid signal peptide; however, on the basis of the intact mass of the observed species as well as its fragmentation pattern, we can infer that the signal peptide is actually cleaved at position 32. The tandem mass spectrum of PA0039, similarly detected with a missing signal peptide, showed robust evidence of a hitherto unannotated disulfide bridge between the cysteines at positions 4 and 42. No fragmentation was observed between these two positions, and the observed intact mass was 2.04 Da lower than predicted [36], matching the intact mass of the disulfide-containing protein form to within 0.1 ppm. PA5178, the second predicted BON domain protein observed in *P. aeruginosa*, was particularly curious; the C-terminal fragment, cleaved off at the boundary between the two predicted domains of the protein, was observed in two colonies subjected to an incubation–cold storage–incubation cycle (see later), though not the third, adjacent colony grown on the same plate and subjected to the same conditions. It was also detected and identified by MS/MS in a colony stored for 3 months at 4 °C, which yielded extremely poor protein mass spectra consistent with extensive protein breakdown. A predicted thermolysin cleavage site was identified by PeptideCutter [38] at the site of observed fragmentation; it is therefore possible that the cleavage is conducted by an as yet unidentified protease and may be functionally relevant. Finally, two examples of ribosomal proteins hitherto inferred from homology [36], L35 (with its N-terminal methionine cleaved) and L36, were also seen.

Sixteen proteins from *P. aeruginosa* were fragmented, and 15 of these were successfully identified. One protein, observed in charge states 6+ and 7+ at *m*/*z* 1237 and *m*/*z* 1060 respectively (measured mass 7419.73 Da), could not be identified by the current method. The only sequence tag generated by ProSight 3.0 from multiple acquired tandem mass spectra consisted of five amino acids, QTAVQ—a much shorter signature than expected of a protein of this size. This observation may suggest a highly modified protein, or one with an unusual structure, and implies that the nonspecificity of the proteome database to this particular strain is not the reason for the lack of identification; however, the repeated detection of this protein in multiple mass spectra suggests it may be linked to cold stress response—it was observed only in colonies that had been stored at 4 °C or room temperature (conditions suboptimal for *P. aeruginosa*, which thrives at 37 °C). Other software packages, such as MSAlign [39], may facilitate the identification of uncharacterized, highly modified proteins.

All proteins selected for fragmentation from *S. aureus* were successfully identified. Five proteins were identified, three of which belong to the phenol-soluble modulin family of small secreted proteins found in virulent staphylococci [40]. δ-Hemolysin, a 26 amino acid peptide that oligomerizes into pore-like structures [41] and subsequently lyses erythrocytes, is one of two well-characterized examples on the list. The other example, phenol-soluble modulin α_4_, is implicated in the lysis of neutrophils [42]; in this particular strain, its existence has so far been inferred only from homology, although in the methicillin-resistant *S. aureus* strain USA300 an identical gene product has been directly observed and studied in detail. A related, predicted protein, SAOUHSC_01135 [43], was also identified; all three retain N-terminal formylmethionine characteristic of this family [40]. SAOUHSC_00845 [44] has been assigned by sequence homology to the previously mentioned UPF0337 family of stress response proteins, detected in all three species. Finally, the predicted protein SAOUHSC_01729 [43], identified here with no modifications, is completely uncharacterized; identical gene products have been found by a standard protein BLAST search in numerous strains of *S. aureus*, as well as one strain of *Staphylococcus haemolyticus*.

Sixteen proteins from *E. coli* (BL21 and K-12) were selected for fragmentation, with 15 successfully identified; the one failure, a 30-kDa protein observed in a single mass spectrum, was due to the poor quality of the MS/MS spectrum. All proteins previously detected in *E. coli* K-12 by direct LESA MS were also detected here. Five of these were selected for MS/MS identification (see Table 1). In addition to these, one previously unseen protein and nine proteins identified by other techniques (five by peptide mass fingerprinting by MALDI-TOF MS, as part of large-scale interaction studies [45, 46], and four by gel electrophoresis [47] and top-down MALDI MS [48, 49]) were identified. Notably, UniProt does not accept the interaction studies mentioned above as sufficient experimental evidence for the existence of the five proteins (YmdF, YciG, YahO, YdfK, and YnaE) that are clearly identified here. YbgS, a protein inferred from homology and shown to display genetic interactions with several partners [50], was detected with its signal peptide cleaved. MS/MS data suggested the presence of a disulfide bond as well as possible deamidation, although insufficient information was available to determine the exact position of the modifications.

The improvements in software for top-down protein analysis play a major part in the method. YdfK, a hypothetical cold shock protein [51], could not be identified in a previous study using the search modes and databases provided by ProSight 2.0 [30]. Highly confident hits with the correct intact mass for this protein were obtained here with use of a custom *E. coli* K-12 proteome database in ProSight 3.0. In addition, YahO was initially identified only by the biomarker search, which takes into account subsequences of the archived proteins as well as their intact forms; the absolute mass search proved, however, sufficient when an updated (2017) database was used. YahO was identified following cleavage of a 21 amino acid sequence peptide from the intact protein, previously inferred from protein sequence [52] and experimentally confirmed here by LESA MS.

Of the proteins observed in streptococci, four were selected for identification: two in *S. pneumoniae* and one each in *S. oralis* and *S. gordonii*. All four were found to be members of the ubiquitous but poorly studied UPF0337 stress response family of proteins; none have thus far been observed in their intact form by other techniques. Their markedly different masses make them suitable as identification markers.

### De Novo Sequencing of an Unknown Protein from an Unknown *Staphylococcus* Species

**Figure 2:**
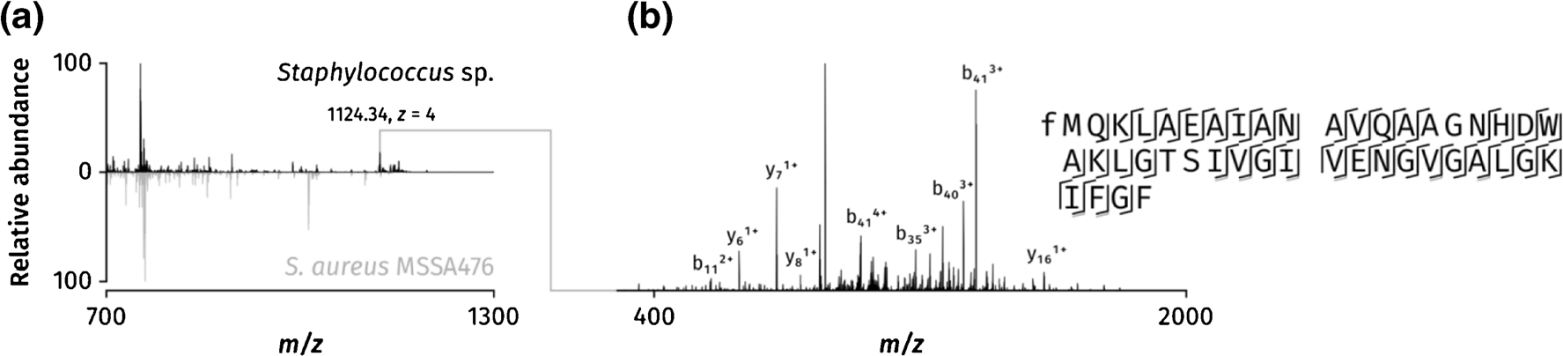
Characterization of the unknown species of *Staphylococcus*. (**a**) Comparison of full LESA mass spectra of *Staphylococcus* sp. and *S. aureus*. (**b**) Collision-induced dissociation mass spectrum of the ion centered at *m*/*z* 1124.34 (4+), identified as a putative phenol-soluble modulin β protein on the basis of de novo sequencing

Thus far, all experiments were conducted on well-characterized species, for which full genome sequences are readily available. The usefulness of LESA MS in bacterial work is, however, not limited to the identification of unknown proteins; with the use of de novo protein sequencing, it can potentially be extended to the characterization of novel microbial species. This capability was demonstrated on the original *Staphylococcus* sample selected for top-down protein analysis and originally thought to be an in-house strain of *S. epidermidis*. In overview, the full mass spectra of this species were dominated by small secreted proteins, similarly to *S. aureus* (Figure 2a). More than 26 peaks were observed, corresponding to at least eight unique protein and peptide masses. On acquisition of tandem mass spectra of several highly abundant protein peaks, however, it was discovered that none of these precisely matched any known *S. epidermidis* proteins. A subsequent search against an all *Staphylococcus* protein database comprising more than 100 strains similarly returned no matches.

To shed light on the true identity of the bacterium, a 4-kDa peptide observed at *m*/*z* ~1124 was analyzed in detail (Figure 2b). Matching of the deconvoluted tandem mass spectrum directly against custom ProSight 3.0 databases derived from a reference *S. epidermidis* proteome placed the unknown protein in the phenol-soluble modulin β family. The strongest hit returned by the software (see Supplementary File 3 for a complete list of candidate sequences and their associated scores) was then used as a guide for de novo sequencing. With this approach, an almost complete sequence was elucidated. Two gaps (AGN and TSI; Figure 2b) were identified in which the nature but not the sequence of the amino acids could be deduced; these were arranged to best match the most closely related homologs found by a standard protein BLAST search. The final sequence is 75% identical to the *S. epidermidis* guide sequence identified by ProSight, 68% identical to the best match returned in the all *Staphylococcus* protein database search (*S. aureus* NCTC 8325, uncharacterized protein SAOUHSC_1135), and 80% identical to a *Staphylococcus capitis* homolog. In the same vein, several partial sequences of the other isolated proteins were acquired, although none were sufficiently complete to allow a meaningful BLAST search. Nevertheless, with some improvements to the software to avoid the most time-consuming step of manual de novo sequencing, as well as to make use of incomplete protein sequences, the ability of this technique to rapidly classify and characterize novel species could be greatly expanded.

### The Influence of Refrigeration

The capability of LESA MS (and any other technique relying on the measurement of protein signal) to identify bacteria has a potential weakness: any variability in the range of the protein peaks observed may introduce uncertainties into the identification, unless this variability is measured and taken into account. The most likely source of such variability in a laboratory or clinical setting is a change in the temperature to which the sample colonies are exposed as a result of transport or storage, prompting changes in protein expression.

On the other hand, should LESA MS detect differences in protein expression under varying conditions or following specific stimuli, it could potentially be used as a tool to shed light on the function of unknown proteins; more generally, it may provide valuable, real-time information on the interactions of bacterial colonies and biofilms and their responses to cues in their environment.

Accordingly, it has previously been demonstrated that the storage of *E. coli* K-12 colonies at 4 °C before analysis has a marked effect on the protein signal in the acquired mass spectra, as compared with mass spectra of colonies sampled immediately after incubation [30].

**Figure 3:**
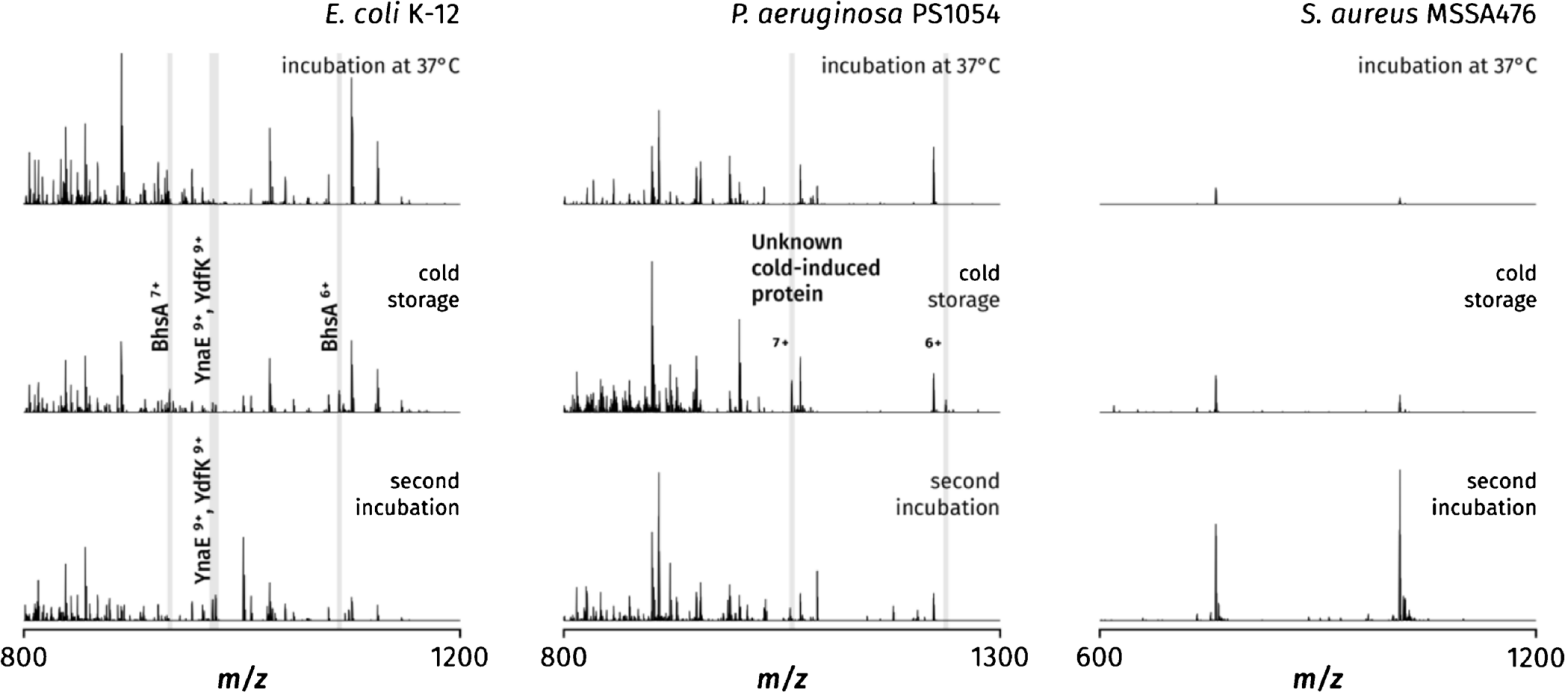
Investigation into the effects of refrigeration on LESA mass spectra of three bacterial species. Each set of three mass spectra corresponds to three colonies grown and sampled from the same agar plate; for each species, the first sampled set of replicates is shown. The solvent system used was 40:60:1 acetonitrile–water–formic acid for *E. coli* and *P. aeruginosa*, and 50:45:5 acetonitrile–water–formic acid for *S. aureus*

Figure 3 shows the results of a systematic investigation into this effect, as observed in the three bacterial species tested, *P. aeruginosa*, *E. coli*, and *S. aureus*. For each species, nine colonies grown on a single Lysogeny broth agar (LBA) plate were subjected to sequential changes in temperature: 37 °C for 24 h (standard incubation protocol), 4 °C for 4 days, and finally 37 °C for 24 h once again; the second incubation step was included to investigate whether any putative changes introduced into the mass spectra by cold storage might be reversible. Following each period, three of the colonies were sampled by LESA MS. Each set of three mass spectra, corresponding to the three periods of incubation or storage, were normalized. Experiments were performed in triplicate, with three identical plates per species, to ensure reproducibility.

Previously, we reported the appearance of a multiple stress response protein, BhsA, in mass spectra of *E. coli* colonies stored at 4 °C before sampling. In the controlled study presented here, as expected from these observations, the BhsA signal was absent in colonies sampled fresh after incubation but prominent peaks corresponding to the 7+ and 6+ charge states of the protein were detected in all colonies sampled following cold storage. Crucially, the peaks disappeared following the second incubation step, confirming that the detection of BhsA in *E. coli* colonies is dependent on the exposure of the colonies to low temperatures immediately before sampling, consistent with its expected expression during cold stress. Two additional peaks corresponding to YnaE and YdfK, the two cold shock proteins known from their transcripts, were seen alongside BhsA following refrigeration—however, unlike BhsA, these proteins remained detectable even after the second incubation. This suggests that refrigeration may, in some cases, lead to persistent changes in the sample that can be detected by LESA MS.

The mass spectra of *P. aeruginosa* displayed behavior similar to that of *E. coli*. Most of the peaks corresponding to proteins remained consistent across all conditions, with additional peaks corresponding to an unknown protein of mass 7419 Da observed in the mass spectra collected immediately following cold storage. The protein, mentioned briefly in the previous section, could not be identified with the current method because of the unusually short sequence tag generated from its tandem mass spectra. Its appearance following refrigeration points to its potential role in cold shock response; we propose that in a similar approach, putative function could be assigned to other unknown proteins following the deliberate exposure of the colonies to particular environmental conditions before sampling. These could include changes in temperature, pH, or exposure to chemical signals and drugs, including antibiotics, which could potentially prompt a resistance response detectable by LESA MS.

The changes observed in the mass spectra of *S. aureus* were different in nature from those seen in the Gram-negative species. No peaks corresponding to putative cold shock proteins were observed. The most abundant species for all conditions remained δ-hemolysin; however, once the set of mass spectra were normalized, it became apparent that the abundance of δ-hemolysin peaks increased over the course of the experiment. The most intense signal was invariably observed following the second incubation. This observation is consistent with the literature suggesting that the highest expression of δ-hemolysin and related, secreted proteins occurs during stationary phase [53].

## Conclusions

We have demonstrated that LESA MS is a powerful tool for the analysis of intact proteins from bacterial colonies. The approach can be applied to Gram-positive bacteria as well as Gram-negative bacteria. The LESA mass spectra were reproducible within species and allowed differentiation of *E. coli*, *P. aeruginosa*, *S. aureus*, *S. pneumoniae*, *S. oralis*, and *S. gordonii*, but it was not possible to differentiate between the two strains of *E. coli* analyzed: K-12 and BL21 mCherry. The effect of refrigeration on the three species was determined. Forty proteins were identified by top-down MS/MS, of which 16 had previously only been predicted on the basis of genome sequencing and had not been observed as proteins and one was entirely novel. Of the total proteins selected for fragmentation and searched against proteome databases with use of ProSight, only two of 41 were not identified, an identification rate of 95%. We have also demonstrated that LESA MS is a suitable tool for the identification of proteins from unknown species.

## Electronic supplementary material

Below is the link to the electronic supplementary material.

Supplementary File 1 presents the outcome of a safety check aiming to determine the viability of bacterial cells extracted by LESA sampling, performed on *Pseudomonas aeruginosa* PS1054 as detailed in the experimental section of the main text. (PPTX 1589 kb)

Supplementary File 2 contains a series of lists providing full, manual fragment assignments for all identified proteins, as listed in Table 1 in the main text; UniProt accession codes and amino acid sequences are included. Fragment assignments for the novel protein sequenced de novo from *Staphylococcus* sp. are also provided. (XLSX 240 kb)

Supplementary File 3 contains a list of phenol soluble modulins β included in the *Staphylococcus epidermidis* database used to provide a rough classification for the protein sequenced de novo from *Staphylococcus* sp.; the corresponding scores returned by ProSight are provided. (DOCX 21 kb)
