# Supplementary material for: Top-Down LESA Mass Spectrometry Protein Analysis of Gram-Positive and Gram-Negative Bacteria
Source: J Am Soc Mass Spectrom. 2017 Jul 5;28(10):2066–77. doi: 10.1007/s13361-017-1718-8 (PMC5594050; doi:10.1007/s13361-017-1718-8)
Supplement: Supplementary file 1 — presents the outcome of a safety check aiming to determine the viability of bacterial cells extracted by LESA sampling, performed on Pseudomonas aeruginosa PS1054 as detailed in the experimental section of the main text. (PPTX 1589 kb) [file 13361_2017_1718_MOESM1_ESM.pptx]

## Slide 1
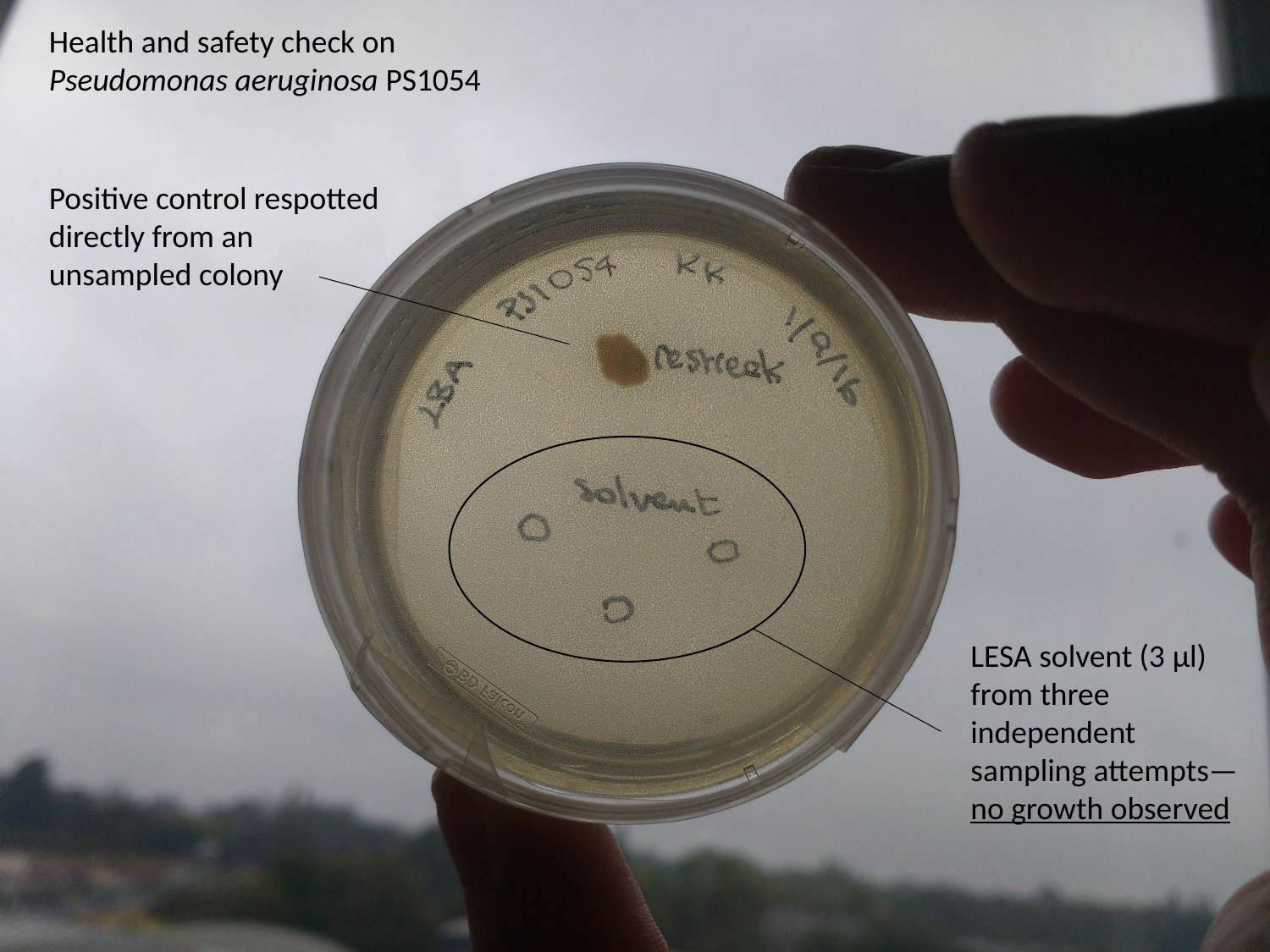

Health and safety check on Pseudomonas aeruginosa PS1054
Positive control respotted directly from an unsampled colony
LESA solvent (3 μl) from three independent sampling attempts—no growth observed
