# Supplementary material for: Top-Down LESA Mass Spectrometry Protein Analysis of Gram-Positive and Gram-Negative Bacteria
Source: J Am Soc Mass Spectrom. 2017 Jul 5;28(10):2066–77. doi: 10.1007/s13361-017-1718-8 (PMC5594050; doi:10.1007/s13361-017-1718-8)
Supplement: Supplementary file 3 — contains a list of phenol soluble modulins β included in the Staphylococcus epidermidis database used to provide a rough classification for the protein sequenced de novo from Staphylococcus sp.; the corresponding scores returned by ProSight are provided. (DOCX 21 kb) [file 13361_2017_1718_MOESM3_ESM.docx]

**Phenol soluble modulins of *Staphylococcus epidermidis* (strain ATCC 35984 / RP62A)**

**Search settings: single protein, fragment tolerance 5 ppm, Δm on**

**Formylation of initial methionine added based on existing literature**

>tr|Q5HQ19|Q5HQ19_STAEQ Phenol soluble modulin beta 1 OS=Staphylococcus epidermidis (strain ATCC 35984 / RP62A) GN=SERP0738 PE=4 SV=1

MSKLAEAIANTVKAAQDQDWTKLGTSIVDIVESGVSVLGKIFGF

| **P Score:** | 1.97E-15 |
| --- | --- |
| **Expectation:** | 1.97E-15 |
| **PDE:** | 31.9000 |

With fM:

| **P Score:** | 4.87E-17 |
| --- | --- |
| **Expectation:** | 4.87E-17 |
| **PDE:** | 32.1000 |

-

>tr|Q5HQ20|Q5HQ20_STAEQ Phenol soluble modulin beta 1 OS=Staphylococcus epidermidis (strain ATCC 35984 / RP62A) GN=SERP0737 PE=4 SV=1

MEQLFDAIRSVVDAGINQDWSQLASGIAGIVENGISVISKLLGQ

| **P Score:** | 0.0421 |
| --- | --- |
| **Expectation:** | 0.0421 |
| **PDE:** | 0.5090 |

With fM:

| **P Score:** | 0.0421 |
| --- | --- |
| **Expectation:** | 0.0421 |
| **PDE:** | 0.5090 |

-

>tr|Q5HKE9|Q5HKE9_STAEQ Phenol soluble modulin beta 1 OS=Staphylococcus epidermidis (strain ATCC 35984 / RP62A) GN=SERP2397 PE=4 SV=1

MEHVSKLGEAIVDTVTAAQAEDGAELAKSIVNIVANAGGIIQDIAHAFGY

| **P Score:** | 0.276 |
| --- | --- |
| **Expectation:** | 0.276 |
| **PDE:** | 0.0629 |

With fM:

| **P Score:** | 1 |
| --- | --- |
| **Expectation:** | 1 |
| **PDE:** | 0.0000 |

-

>tr|Q5HQ21|Q5HQ21_STAEQ Phenol soluble modulin beta 1 OS=Staphylococcus epidermidis (strain ATCC 35984 / RP62A) GN=SERP0736 PE=4 SV=1

MELLTHLGVLIMKLFNAFKDILEAAITNDGTQLGASIVNIIESSVDMVNRFLGN

| **P Score:** | 0.276 |
| --- | --- |
| **Expectation:** | 0.276 |
| **PDE:** | 0.1250 |

With fM:

| **P Score:** | 1 |
| --- | --- |
| **Expectation:** | 1 |
| **PDE:** | 0.0000 |

-

>tr|Q5HKE6|Q5HKE6_STAEQ Phenol soluble modulin beta 1 OS=Staphylococcus epidermidis (strain ATCC 35984 / RP62A) GN=SERP2400 PE=4 SV=1

MEHVSKLAEAIANTVSAAQAEDGAELAKSIVNIVANAGGIIQDIAHAFGY

| **P Score:** | 1 |
| --- | --- |
| **Expectation:** | 1 |
| **PDE:** | 0.0000 |

With fM:

| **P Score:** | 1 |
| --- | --- |
| **Expectation:** | 1 |
| **PDE:** | 0.0000 |
